# Spinosad resistance affects biological parameters of *Musca domestica* Linnaeus

**DOI:** 10.1038/s41598-018-32445-8

**Published:** 2018-09-19

**Authors:** Hafiz Azhar Ali Khan

**Affiliations:** Institute of Agricultural Sciences, University of the Punjab, Lahore, Pakistan

## Abstract

*Musca domestica* is one of the major cosmopolitan insect pests of public health importance. Spinosad is considered an eco-friendly insecticide used for the management of *M*. *domestica* and other pests of significant concern. Cases of resistance against spinosad in *M*. *domestica* have been reported from some parts of the world; however, there are no reports of any negative effects of spinosad resistance on the fitness/biological parameters of *M*. *domestica*. To investigate fitness costs, a near isogenic *M*. *domestica* resistant strain (Spin-R) was constructed using Spin-UNSEL-susceptible and Spin-SEL-resistant strains sharing a close genetic background. We found significantly reduced rates of adult eclosion, fecundity, egg hatching, survival, and lengthened developmental time in the Spin-R strain. Moreover, the values of different fitness parameters like biotic potential, mean relative growth rate, intrinsic rate of natural increase, and net reproductive rate, were also significantly reduced in the Spin-R strain, which reflect fitness costs most probably linked with spinosad resistance. The presence of fitness costs suggests likely instability of resistance to spinosad in *M*. *domestica*, which can be reverted by relaxing spinosad selection pressure and rotation with alternate insecticides. The wise use of insecticides will ultimately help to manage resistance in this pest and minimize environmental pollution.

## Introduction

*Musca domestica* Linnaeus is an economic pest of animal agriculture and public health which grows rapidly in unhygienic environmental conditions. It is not only a source of nuisance but also plays a role in disease transmissions through its rapid expansion and forcing the affected communities to rely on the heavy use of various insecticides^1^. This practice has been adopted worldwide with the resulting evolution of resistance in *M*. *domestica* against a number of insecticides^2–6^. As a consequence, the affected people shift from the recommended dose to over-dosage of insecticides with the ultimate negative impact on the environment and public health^7^. Therefore, the search for new insecticides to manage resistant insects and to develop resistance management strategies is of prime importance^8^. Among these relatively new insecticides, spinosad (derivative of a soil actinomycete *Saccharopolyspora spinosa*), has been considered safe due to very low toxicity towards mammals and other non-target organisms^9–12^. However, different insect pests like *Aedes albopictus* (Skuse)^13^, *Drosophila melanogaster* Meigen^14^, *Plutella xylostella* L.^15^, *Helicoverpa armigera* (Hübner)^16^, *Tribolium castaneum*^17^ and *M*. *domestica*^6,18^ have developed resistance against spinosad in some parts of the world.

There are some important factors which contribute to an insect pest developing resistance to a particular insecticide. These factors include the performance of biological parameters of a species in the presence of insecticide selection pressure, fitness costs, frequency of resistance allele(s), pest management practices and population dynamics^19,20^. Among these factors, fitness costs and the performance of biological parameters associated with resistance to a particular insecticide seem to vary considerably among species and insecticides^21^. The fitness costs i.e., lengthened developmental time, reduced fecundity and survival, have been widely assumed to be linked with mutations that confer insecticide resistance^22^. Generally speaking, fitness costs can be enhanced under stressful environments like the presence of insecticide selection pressure^23^. The poor performance of biological parameters and fitness costs due to insecticide resistance contribute to limiting an increase in resistance alleles^24^. Similarly, in the presence of these factors in a population, the stability of resistance allele(s), and subsequent transmissions to the next generations could be prevented by integrated management practices along with the wise use of insecticides^24,25^.

Previously, we have reported low levels of resistance to spinosad in *M*. *domestica* from Pakistan^26^. Thereafter, in order to understand the nature of resistance, we have reported on the inheritance and preliminary mechanism of spinosad resistance in *M*. *domestica* under laboratory selections^27^. The resistance was inherited autosomally, incompletely dominant and governed by more than one gene. In addition, absence of metabolic mechanism of resistance was reported in the resistant strain suggesting the possibility of an altered target site mechanism^18^. However, resistance development to spinosad was unstable and declined rapidly when spinosad selection pressure was lifted, thus forming the basis of a hypothesis that decline in resistance could be associated with fitness costs. There are a number of reported cases where high fitness costs were linked with spinosad resistance development in different insect species^28–31^. However, this information is sparse in the case of *M*. *domestica*. Studies on fitness costs in resistant populations are important for devising an effective resistance management strategy, and to prevent the misuse of insecticides^32^. Such studies ultimately help to minimize environmental pollution and negative impacts on public health. Therefore, the purpose of the present study was to determine the effects of spinosad resistance on different fitness/biological parameters of *M*. *domestica*. For this purpose, a near isogenic strain of *M*. *domestica* resistant to spinosad was established since such an approach is generally regarded asthe best for fitness assessments^33^.

## Results

The median lethal concentrations (LC_50s_) for Spin-UNSEL and Spin-SEL strains were 1.93 and 122.56 µg/ml, respectively. The Spin-SEL strain showed 155.14 fold resistance ratio (RR) to spinosad when compared with the Lab-susceptible strain^27^ (Table 1). In the process of isolating the near isogenic line with spinosad resistance, the RR values in the BC_n_F1 progeny decreased significantly after backcrossing with the susceptible recurrent parent; however, these values increased in the BC_n_F2 progeny after self-breeding. The LC_50_ value increased to 143.86 µg/ml in the BC5F2 progeny, and did not fluctuate in the next progenies (BC5F3, or BC5F4) based on the overlapping 95% CIs, suggesting that the near isogenic line (Spin-R) with spinosad resistance was established (Table 1).

**Table 1:** Toxicity of spinosad against different strains of *Musca domestica*.

Strain	LC_50_ (95% CI) (µg/ml)	Fit of probit line				RR**
		Slope (SE)	χ^2^	df	P	
Spin-UNSEL	1.93 (1.60–2.40)	2.14 (0.23)	1.99	3	0.57	
Spin-SEL	122.56 (104.25–144.05)*	2.46 (0.24)	1.01	3	0.80	63.50
BC1F1	21.75 (18.01–26.82)	1.91 (0.18)	1.77	4	0.78	11.27
BC1F2	51.86 (41.12–71.79)	2.04 (0.27)	0.27	3	0.97	26.87
BC2F1	32.56 (25.85–43.43)	1.65 (0.19)	1.05	4	0.90	16.87
BC2F2	75.86 (61.19–99.93)	1.90 (0.23)	1.25	3	0.74	39.30
BC3F1	20.07 (16.41–24.88)	1.81 (0.20)	3.34	3	0.34	10.40
BC3F2	95.36 (80.87–113.95)	2.38 (0.24)	1.84	3	0.61	49.93
BC4F1	34.12 (26.63–47.27)	1.54 (0.21)	2.42	3	0.49	17.68
BC4F2	127.36 (106.38–157.31)	2.24 (0.24)	2.36	3	0.51	65.99
BC5F1	28.98 (22.87–38.88)	1.55 (0.20)	3.33	3	0.34	15.02
BC5F2	143.86 (118.95–181.51)	2.17 (0.25)	3.42	3	0.33	74.54
BC5F3	138.17 (113.31–176.29)	2.04 (0.23)	2.12	3	0.55	71.59
BC5F4 (Spin-R)	151.28 (123.66–194.91)	2.06 (0.24)	2.69	3	0.43	78.38

*LC_50_ values and corresponding statistics reported previously^27^.

**RR = LC_50_ of the Spin-SEL or BC_n_F_n_/LC_50_ of the Spin-UNSEL strain of *M*. *domestica*.

^£^number exposed.

The comparison of various biological parameters of Spin-UNSEL, Spin-SEL and Spin-R strains revealed significant differences (Table 2), that might reflect the presence of fitness costs in *M*. *domestica*. For instance, the survival of the Spin-R strain at the larval stage was significantly lower (65 ± 4.86%) compared with the Spin-SEL (71.33 ± 2.07%) and Spin-UNSEL (87.67 ± 1.80%) strains (F = 33.6; df = 2,12; *p* < 0.01). The larval stage took more time to complete in the Spin-R strain (6.20 ± 0.57days) as compared with the Spin-SEL (5.20 ± 0.12days) and Spin-UNSEL (4.40 ± 0.19days) strains (F = 21.2; df = 2,12; *p* < 0.05). Relatively heavier pupae were observed in the case of Spin-UNSEL (19.90 ± 0.56 mg) compared with the Spin-SEL (17.54 ± 0.21 mg) and Spin-R (16.08 ± 0.32 mg) strains (F = 29.9; df = 2,12; *p* < 0.01). The pupae of the Spin-UNSEL strain developed faster (4.80 ± 0.27days) than those of the Spin-SEL (5.70 ± 0.27days) and Spin-R (6.90 ± 0.42days) strains. In short, the total development time (from egg hatching to adult formation) lengthened in case of the Spin-R strain (13.30 ± 0.57days) compared with the Spin-SEL (12.20 ± 0.27days) and Spin-UNSEL (10.20 ± 0.45days) strains (F = 12.35; df = 2,12; *p* < 0.001).

**Table 2:** Comparison of biological parameters of different strains of *M*. *domestica*.

Biological parameter	Strain			*p* value
	Spin-UNSEL	Spin-SEL	Spin-R	
Neonates	300*	300*	300*	
Survival at larval stage (%)	87.67 ± 1.80a	71.33 ± 2.07b	65.00 ± 4.86c	<0.01
Larval duration (d)	4.40 ± 0.19c	5.20 ± 0.12b	6.20 ± 0.57 a	<0.05
Pupal weight (mg)	19.90 ± 0.56a	17.54 ± 0.21b	16.08 ± 0.32c	<0.01
Pupal duration (d)	4.80 ± 0.27c	5.70 ± 0.27b	6.90 ± 0.42a	<0.01
Total developmental time (d)	10.20 ± 0.45c	12.20 ± 0.27b	13.30 ± 0.57a	<0.001
Adult eclosion (%)	82.67 ± 1.45a	75.00 ± 1.75b	65.66 ± 3.65c	<0.05
Female ratio (%)	49.98 ± 3.13a	45.75 ± 1.54b	47.19 ± 1.87ab	<0.05
Fecundity (lifetime eggs/female)	340.80 ± 11.37a	207.20 ± 8.17b	198.80 ± 9.56 b	<0.001
Egg hatching (%)	81.20 ± 2.28a	72.00 ± 3.08b	66.20 ± 3.03c	<0.001
Intrinsic rate of natural increase (*r*_m_)	0.47 ± 0.02a	0.33 ± 0.01b	0.28 ± 0.01c	<0.01
Mean Relative Growth Rate (MRGR)	1.41 ± 0.06a	1.18 ± 0.02b	1.19 ± 0.06b	<0.05
Biotic potential	0.24 ± 0.004a	0.19 ± 0.001b	0.17 ± 0.01c	<0.001
Net reproductive rate (*R*_o_)	115.88 ± 9.17a	56.81 ± 4.44b	43.16 ± 2.72c	<0.001
Relative fitness (w)	1.00	0.49	0.37	

*Sum of five replicates; *n* = 60 neonates per replicate.

Means sharing different letters (a, b or c) within a row are significantly different.

Besides developmental time, other biological parameters were also significantly different between the studied strains of *M*. *domestica*. For example, 82.67% pupae of the Spin-UNSEL strain were successfully eclosed to adults as compared to 75 and 65% in the case of Spin-SEL and Spin-R pupae, respectively (F = 27.8; df = 2,12; *p* < 0.05). Comparison of the resultant adults revealed a relatively higher number of female *M*. *domestica* in the Spin-UNSEL strain (49.98 ± 3.13%) followed by the Spin-R and Spin-SEL strains (F = 4.41; df = 2,12; *p* < 0.05). Moreover, resultant female *M*. *domestica* of the Spin-UNSEL strain produced a higher number of eggs in their lifetime (340.80 ± 11.37) compared with the Spin-SEL and Spin-R strains (F = 545; df = 2,12; *p* < 0.001), and the proportion of egg hatching also showed the same trend. Regarding survival at the adult stage, the adults of the Spin-UNSEL strain lived significantly longer compared with the Spin-SEL and Spin-R strains (Table 2).

The comparison of fitness parameters including the intrinsic rate of natural increase (*r*_m_), biotic potential (bp), mean relative growth rate (MRGR), net reproductive rate (*R*_o_), and relative fitness also revealed significant differences among all the strains. The values of all of the above fitness parameters were significantly higher in the Spin-UNSEL strain than those of the Spin-SEL and Spin-R strains (F = 198; df = 2,12; *p* < 0.01 for *r*_m_, F = 8.97; df = 2,12; *p* < 0.05 for MRGR, F = 120; df = 2,12; *p* < 0.001 for bp, and F = 202; df = 2,12; *p* < 0.001 for *R*_o_, Table 2).

## Discussion

Environmental contamination resulting from the excessive use of pesticides in agriculture and health sectors can exert strong selection pressure on the exposed populations. Resultantly, survival of the fittest phenotypes with the ability to combat contaminations may arise as a consequence of evolution of resistance to the environmental contaminants^1^. Such directional evolutions in the selected populations are usually expected to have detrimental effects on fitness in the absence of environmental contaminants or selection pressure, thus constrain the evolution of resistance^34^. For example, worker honey bees reared in a pesticide-contaminated brood were found with lower survivorship, lengthened developmental time and higher brood mortality rates compared with bees reared in relatively uncontaminated brood, leading to the assumption that the bees in the contaminated brood were less fit and unable to evolve resistance against pesticides^35^.

The present study was based on the effects of spinosad resistance development on biological parameters in *M*. *domestica*. In our previous work^27^, the Spin-SEL strain showed rapid development of resistance to spinosad under laboratory selections, but resistance was unstable when the selected strain was reared without spinosad. Based on the unstable nature of resistance, it was hypothesized that fitness costs were associated with resistance in the selected strain of *M*. *domestica*. In the present study a near isogenic Spin-R strain was established to confirm the presence of fitness costs and the results have further strengthened the above hypothesis. The comparison of biological parameters of resistant and susceptible strains is a logical approach to evaluate fitness costs; however, misinterpretation of the fitness data may occur owing to genetic background variations between strains^36^. Therefore, in the present study, the near isogenic line of *M*. *domestica* resistant to spinosad was established. Theoretically speaking, the genetic variations between recurrent parental strain and the near isogenic line would only be in a limited number of genes, including the selected gene of interest^37^.

In the present work, the Spin-R strain was compared with Spin-SEL and Spin-UNSEL in terms of development from the egg stage to the adult formation, and in the performance of subsequent adults (fecundity, egg hatching, male to female ratio and survival). The results revealed significant differences in the performance of all the studied biological parameters among the studied strains which showed fitness costs associated with spinosad resistance. The Spin-R strain took significantly more time at the larval stage, which did not translate into heavier pupae compared with Spin-SEL and Spin-UNSEL. The Spin-R strain took more time to develop into adults as compared with Spin-SEL and Spin-UNSEL. Moreover, fecundity, egg hatching and survival at adulthood also decreased significantly in the Spin-R strain. Therefore, spinosad resistance in the Spin-R strain of *M*. *domestica* probably imposes direct fitness costs thus suggesting a natural compromise in the distribution of resources between biological parameters and resistance development allele(s) of the selected strain^38^.

Insecticide resistance mechanisms (e.g., target site or metabolic) could be responsible for inducing fitness costs in the Spin-R strain of *M*. *domestica*. Previously, it has been reported in different insect species that exon skipping^39^, point mutations^40^, or the production of truncated proteins^41^ might be responsible for spinosad resistance in the nicotinic acetylcholine receptor site and associated fitness costs. Similarly, for metabolic resistance mechanism, if the production of detoxifying enzyme is costly, then resistant individuals would not produce such enzymes in the absence of insecticide selection pressure^22^. Overexpression of cytochrome P450 genes has been reported in a spinosad resistant strain (791spin)^42^ and a Danish field strain (791a)^43^ of *M*. *domestica*. However, a high level of spinosad resistance in of *M*. *domestica* are not associated with P450 mediated mechanisms^18,44^. Similarly, resistance to spinosad in our Spin-SEL strain of *M*. *domestica* was unchanged upon pretreatment with an enzyme inhibitor PBO which pointed to the likely absence of P450 metabolic resistance mechanism^27^. However, further studies are needed to confirm the exact mechanism of spinosad resistance in the Spin-SEL strain and associated fitness costs.

Fitness costs have been widely assumed to be linked with mutations that confer insecticide resistance^22^. As a consequence of these mutations, the loci within the genome of resistant insects may act as ‘modifiers’ to decrease fitness costs in the absence of pesticide selection pressure^45^. Theoretically speaking, target-site and metabolic-based pesticide resistance mechanisms could induce fitness costs in the stressed phenotypes^22^. Modifications in the target-site can affect fitness of resistant insects, particularly in situations where target-site substitution is essential for the viability of resistant individuals. Any molecular alteration in the target-site may cause pleiotropic effects on biological parameters which ultimately affect the resistant population’s survival and reproductive success. Metabolic-based mechanisms are based on the theory that the phenotypes in the contaminated or stressed environments consume more energy for maintaining the defense mechanism (immune system, enzymes, etc.) against the environmental stress rather than enhancing fitness components^34^. For instance, in the case of insecticide resistance, measurement of energetic resources like glucose, glycogen and lipids in insecticide resistant mosquitoes (*Culex pipiens* Linnaeus) revealed that the resistant mosquitoes had 30% less such resources compared to their susceptible counterparts^46^. Therefore, fitness costs could be increased under stressful environments such as the presence of insecticide selection pressure^23^. Moreover, if the allele(s) causing insecticide resistance are rare in a population, the development and stability of resistance to a particular insecticide would depend on the relative fitness between the resistant strain and its susceptible counterpart^33^. In this case resistant allele(a) are present in heterozygotes at low frequencies, and if fitness costs are dominant, resistance will develop slowly and often fail to be maintained in the absence of insecticide selection pressure^20,38^. Relatively reduced fitness and weak performance of life history traits as a consequence of spinosad resistance have been reported in different insect pests like *Chrysoperla carnea* (Stephens)^29^, *H*. *armigera*^16^, *Heliothis virescens* (Fabricius)^31^, and *P*. *xylostella*^28,30^. However, the lack of negative effects on biological parameters as a result of spinosad resistance has been reported in *Frankliniella occidentalis* (Pergande)^47^. This indicates that the fitness costs due to spinosad resistance depend on insect pest species in question and/or the mechanism of resistance. However, this information was sparse in case of *M*. *domestica*. The Spin-R strain of *M*. *domestica* proved less fit than the Spin-SEL and Spin-UNSEL strain based on the results of different biological parameters and fitness parameters. For instance, survival at the larval stage of the Spin-UNSEL strain was significantly higher compared with the Spin-R strain. The larval stage took more time to complete in the Spin-R strain in comparison to the Spin-UNSEL strain. Relatively heavier pupae were observed in case of the Spin-UNSEL strain compared with the Spin-R and Spin-SEL strains. The pupae of the Spin-UNSEL strain developed faster than those of the Spin-R strain. In short, the total development time lengthened in case of the Spin-R and Spin-SEL strains. The results of lengthened developmental time in the Spin-R strain are in agreement with those reported for *P*. *xylostella*^28^ where the spinosad selected strain took more time to convert into adults compared with its unselected counterpart strain. The Spin-R strain of *M*. *domestica* took more time at larval stage than the Spin-UNSEL strain, but it failed to produce heavier/larger pupae. Similarly, the spinosad selected larvae of the *Heliothis virescens* also resulted into weaker pupae when compared with those of the susceptible strain^31^. In addition, reduced fecundity, egg hatching, and survival at adulthood point to the fact that the spinosad selection significantly affected the performance of biological parameters in *M*. *domestica*. The biological parameters of resistant insects usually show a poorer performance than those of their susceptible counterparts^29^. The most probable reason for this phenomenon is the fact that the pesticide resistant insects face decline in their energy level and hence are less fit in their environment^48^.

The values of fitness parameters like MRGR, bp, *r*_m_ and *Ro* of the Spin-SEL strain were also significantly reduced. These parameters indicate the potential of a certain population to increase under given environmental conditions^49,50^. The reduced rates of all these parameters in the Spin-R strain clearly demonstrate the presence of the fitness costs phenomenon. In this case, fitness costs is advantageous in terms of managing insecticide resistance since the removal of selection pressure will likely cause a decrease in the number of resistance allele(s)^29,38^, and ultimately reversion of insecticide resistance. This has already been reported in our previous work in which the Spin-SEL strain showed reversion of spinosad resistance when the selection pressure was lifted^27^.

In conclusion, the data of the present study demonstrate that fitness costs are most probably associated with spinosad resistance in the Spin-R strain of *M*. *domestica*. We found significantly reduced rates of adult eclosion, fecundity, egg hatching, survival, and lengthened developmental time in the Spin-R strain. Moreover, the values of different fitness parameters like biotic potential, mean relative growth rate, intrinsic rate of natural increase, and net reproductive rate, were also significantly reduced in the Spin-R strain, which reflect fitness costs most probably linked with spinosad resistance. The presence of fitness costs suggests likely instability of resistance to spinosad in *M*. *domestica* which can be reverted by relaxing spinosad selection pressure and rotation with alternate insecticides. The wise use of insecticides will ultimately help to manage resistance in this pest and minimize environmental pollution. Therefore, combined with our previous findings^27^, spinosad resistance in *M*. *domestica* could be managed by rotational use of insecticides. This will help to manage resistant insects effectively and minimize environmental pollution.

## Materials and Methods

### *Musca domestica* strains

A field strain of *M*. *domestica* was collected from Lahore (31° 32′59 N; 74° 20′37 E) and divided into two sub strains. One sub strain was kept unselected and reared up to 10 generations without exposure to any insecticide, and this strain was designated as “Spin-UNSEL”. The other sub strain was cultured under spinosad selection pressure for 10 consecutive generations and resulted in a 155 fold resistance development compared with the laboratory susceptible strain (Lab-susceptible)^27^. This sub strain was designated as “Spin-SEL”. A near isogenic line (Spin-R) with spinosad resistance was established following the methodology of Horikoshi, *et al*.^36^. In short, the Spin-R strain was derived by repeatedly backcrossing Spin-SEL females with Spin-UNSEL males. The offspring of the backcrosses were referred to as the BC_n_F1 progeny (n = number of backcrosses). The self-bred BC_n_F1 progeny yielded BC_n_F2 progeny which was selected with spinosad using the concentration level to cause 70% mortality (i.e., LC_70_), and then the surviving females were backcrossed to Spin-UNSEL males. The detailed methodology for selection experiment and bioassays has already been reported previously^27^. The selection bioassays and backcrossing were completed when the LC_50_ values of the self-bred progenies BC_n_F2, BC_n_F3, and BC_n_F4 had become stable. All the strains were maintained under laboratory conditions as described previously^26,51^.

### Survival and development of Spin-UNSEL, Spin-SEL and Spin-R strains

Survival and development was checked by following the work of Khan, *et al*.^50^ with a few modifications. Briefly, batches of 60 first instar larvae (<12 h old) of Spin-UNSEL, Spin-SEL and Spin-R strains were maintained on larval media under the above laboratory conditions. These batches were replicated five times to study fitness parameters, and the total number of larvae studied per strain was 300. The growth and survival of the tested larvae were checked twice daily. The parameters recorded were: duration of the larval (1^st^ to 3^rd^ instar) and pupal stages, survival at larval and pupal stages, and total developmental time from hatching to adult formation.

### Fecundity, egg hatching and survival at adulthood

To compare the number of eggs laid, egg hatching and survival at the adulthood stage, five pairs of all the strains (<24 h old) each were kept in separate wooden mesh cages (30 × 30 × 30 cm). The cages were provided with adult food, and larval medium for egg laying and immature development. The cages were kept under the same laboratory conditions as stated earlier. The cages were observed twice daily to count the number of eggs laid, eggs hatched and survival of the adults till the mortality of each pair.

### Fitness parameters

In order to calculate the mean relative growth rate (MRGR), 1^st^ instar larvae (n = 50) of all the strains were taken from their respective cages, weighed, and divided into five equal batches. Each batch was reared separately in a 250 ml glass beaker containing 100 g larval medium. After the completion of the larval stage, pupae were removed and weighed to calculate MRGR according to the following formula^52,53^:

1 $${\rm{MRGR}}=\frac{[\,\mathrm{ln}\,.({\rm{pupal}}\,{\rm{weight}})-\,\mathrm{ln}\,.({\rm{initial}}\,{\rm{larval}}\,{\rm{weight}})]}{{\rm{Time}}\,{\rm{from}}\,{\rm{the}}\,{\rm{larval}}\,{\rm{stage}}\,{\rm{to}}\,{\rm{the}}\,{\rm{pupal}}\,{\rm{stage}}\,({\rm{T}})}$$

Net reproductive rate (*Ro*), intrinsic rate of natural increase (*r*_*m*_) and biotic potential (bp) were calculated as described previously^53^.

Biotic potential was determined by dividing the log fecundity with total developmental time^50,53^, and relative fitness (w) was calculated by dividing *R*_o_ of Spin-SEL or Spin-R strain with *R*_o_ of Spin-UNSEL strain.

To compare each biological parameter of the Spin-SEL, Spin-UNSEL and Spin-R strain, the data were analyzed by the multivariate analysis of variance (MANOVA) and means were compared by Tukey’s HSD test.

### Ethical statement

The study/bioassay protocols used against *M*. *domestica* were performed according to the standard guidelines and regulations and approved by the institutional research project evaluation committee.

## References

[1] Khan HAA, Akram W (2017). Cyromazine resistance in a field strain of house flies, Musca domestica L.: Resistance risk assessment and bio-chemical mechanism. Chemosphere.

[2] Kaufman PE, Nunez SC, Mann RS, Geden CJ, Scharf ME (2010). Nicotinoid and pyrethroid insecticide resistance in houseflies (Diptera: Muscidae) collected from Florida dairies. Pest Management Science.

[3] Khan HA, Akram W, Shad SA (2013). Resistance to conventional insecticides in Pakistani populations of Musca domestica L. (Diptera: Muscidae): a potential ectoparasite of dairy animals. Ecotoxicology.

[4] Khan HA, Akram W, Iqbal J, Naeem-Ullah U (2015). Thiamethoxam Resistance in the House Fly, Musca domestica L.: Current Status, Resistance Selection, Cross-Resistance Potential and Possible Biochemical Mechanisms. Plos One.

[5] Scott JG, Alefantis TG, Kaufman PE, Rutz DA (2000). Insecticide resistance in house flies from caged‐layer poultry facilities. Pest Management Science.

[6] Markussen MD, Kristensen M (2012). Spinosad resistance in female Musca domestica L. from a field-derived population. Pest Management Science.

[7] Hemingway J, Ranson H (2000). Insecticide resistance in insect vectors of human disease. Annu. Rev. Entomol..

[8] Ali Khan HA, Akram W, Lee S (2018). Resistance to Selected Pyrethroid Insecticides in the Malaria Mosquito, Anopheles stephensi (Diptera: Culicidae), From Punjab, Pakistan. J. Med. Entomol..

[9] Sparks, T. *et al*. In *Beltwide Cotton Conferences* (*USA*) (1997).

[10] Salgado VL (1998). Studies on the mode of action of spinosad: insect symptoms and physiological correlates. Pestic. Biochem. Physiol..

[11] Khan HAA, Akram W (2018). Trichlorfon and spinosad resistance survey and preliminary determination of the resistance mechanism in Pakistani field strains of Bactrocera dorsalis. Scientific Reports (Nature Publisher Group).

[12] Khan T, Shahid AA, Khan HAA (2016). Could biorational insecticides be used in the management of aflatoxigenic Aspergillus parasiticus and its insect vectors in stored wheat?. PeerJ.

[13] Khan HAA, Akram W, Shehzad K, Shaalan EA (2011). First report of field evolved resistance to agrochemicals in dengue mosquito, Aedes albopictus (Diptera: Culicidae), from Pakistan. Parasit Vectors.

[14] Perry T, McKenzie JA, Batterham P (2007). A Dα6 knockout strain of Drosophila melanogaster confers a high level of resistance to spinosad. Insect Biochem. Mol. Biol..

[15] Zhao J (2006). Monitoring of diamondback moth (Lepidoptera: Plutellidae) resistance to spinosad, indoxacarb, and emamectin benzoate. J. Econ. Entomol..

[16] Wang D, Qiu X, Ren X, Niu F, Wang K (2009). Resistance selection and biochemical characterization of spinosad resistance in Helicoverpa armigera (Hübner)(Lepidoptera: Noctuidae). Pestic. Biochem. Physiol..

[17] Khan HafizAzharAli (2018). Monitoring Susceptibility to Spinosad in Three Major Stored-Product Insect Species from Punjab, Pakistan. Pakistan. Pakistan Journal of Zoology.

[18] Shi J, Zhang L, Gao X (2011). Characterisation of spinosad resistance in the housefly Musca domestica (Diptera: Muscidae). Pest Management Science.

[19] Gould F (1998). Sustainability of transgenic insecticidal cultivars: integrating pest genetics and ecology. Annu. Rev. Entomol..

[20] Carriére Y, Tabashnik B (2001). Reversing insect adaptation to transgenic insecticidal plants. Proceedings of the Royal Society of London B: Biological Sciences.

[21] Roush, R. T. & Daly, J. C. In *Pesticide resistance in arthropods* 97–152 (Springer, 1990).

[22] ffrench-Constant RH, Bass C (2017). Does resistance really carry a fitness cost?. Current Opinion in Insect Science.

[23] Raymond B, Sayyed AH, Wright DJ (2007). Host plant and population determine the fitness costs of resistance to Bacillus thuringiensis. Biology Letters.

[24] Basit M, Sayyed AH, Saeed S, Saleem MA (2012). Lack of fitness costs associated with acetamiprid resistance in Bemisia tabaci (Hemiptera: Aleyrodidae). J. Econ. Entomol..

[25] Bates SL (2005). Evaluation of a chemically inducible promoter for developing a within-plant refuge for resistance management. J. Econ. Entomol..

[26] Khan HA, Shad SA, Akram W (2013). Resistance to new chemical insecticides in the house fly, Musca domestica L., from dairies in Punjab, Pakistan. Parasitol. Res..

[27] Khan HA, Akram W, Shad SA (2014). Genetics, cross-resistance and mechanism of resistance to spinosad in a field strain of Musca domestica L. (Diptera: Muscidae). Acta Trop..

[28] Sayyed AH, Saeed S, Crickmore N (2008). Genetic, biochemical, and physiological characterization of spinosad resistance in Plutella xylostella (Lepidoptera: Plutellidae). J. Econ. Entomol..

[29] Abbas N (2014). Fitness cost and realized heritability of resistance to spinosad in Chrysoperla carnea (Neuroptera: Chrysopidae). Bull. Entomol. Res..

[30] Li Z, Liu S, Liu Y, Ye G (2007). Temperature-related fitness costs of resistance to spinosad in the diamondback moth, Plutella xylostella (Lepidoptera: Plutelidae). Bull. Entomol. Res..

[31] Wyss CF, Young HP, Shukla J, Roe RM (2003). Biology and genetics of a laboratory strain of the tobacco budworm, Heliothis virescens (Lepidoptera: Noctuidae), highly resistant to spinosad. Crop Protection.

[32] Gassmann, A. J., Carrière, Y. & Tabashnik, B. E. Fitness costs of insect resistance to Bacillus thuringiensis. *Annu*. *Rev*. *Entomol*. **54** (2009). 10.1146/annurev.ento.54.110807.090518 19067630

[33] Roush RT, McKenzie JA (1987). Ecological genetics of insecticide and acaricide resistance. Annu. Rev. Entomol..

[34] Xie L, Klerks PL (2004). Fitness cost of resistance to cadmium in the least killifish (Heterandria formosa). Environmental Toxicology and Chemistry.

[35] Wu JY, Anelli CM, Sheppard WS (2011). Sub-lethal effects of pesticide residues in brood comb on worker honey bee (Apis mellifera) development and longevity. Plos One.

[36] Horikoshi RJ (2015). Near-isogenic Cry1F-resistant strain of Spodoptera frugiperda (Lepidoptera: Noctuidae) to investigate fitness cost associated with resistance in Brazil. J. Econ. Entomol..

[37] Zhu X (2015). Construction and characterisation of near‐isogenic Plutella xylostella (Lepidoptera: Plutellidae) strains resistant to Cry1Ac toxin. Pest Manage. Sci..

[38] Sayyed AH, Ahmad M, Crickmore N (2008). Fitness costs limit the development of resistance to indoxacarb and deltamethrin in Heliothis virescens (Lepidoptera: Noctuidae). J. Econ. Entomol..

[39] Berger M (2016). Insecticide resistance mediated by an exon skipping event. Mol. Ecol..

[40] Puinean AM, Lansdell SJ, Collins T, Bielza P, Millar NS (2013). A nicotinic acetylcholine receptor transmembrane point mutation (G275E) associated with resistance to spinosad in Frankliniella occidentalis. J. Neurochem..

[41] Hsu J-C (2012). Truncated transcripts of nicotinic acetylcholine subunit gene Bdα6 are associated with spinosad resistance in Bactrocera dorsalis. Insect Biochem. Mol. Biol..

[42] Højland DH, Jensen K-MV, Kristensen M (2014). Expression of xenobiotic metabolizing cytochrome P450 genes in a spinosad-resistant Musca domestica L. strain. Plos One.

[43] Markussen MD, Kristensen M (2012). Spinosad resistance in female Musca domestica L. from a field‐derived population. Pest Management Science.

[44] Shono T, Scott JG (2003). Spinosad resistance in the housefly, Musca domestica, is due to a recessive factor on autosome 1. Pestic. Biochem. Physiol..

[45] Fisher, R. A. *The genetical theory of natural selection: a complete variorum edition*. (Oxford University Press, 1999).

[46] Rivero A, Magaud A, Nicot A, Vézilier J (2011). Energetic cost of insecticide resistance in Culex pipiens mosquitoes. J. Med. Entomol..

[47] Bielza P, Quinto V, Gravalos C, Abellan J, Fernandez E (2008). Lack of fitness costs of insecticide resistance in the western flower thrips (Thysanoptera: Thripidae). J. Econ. Entomol..

[48] Kliot A, Ghanim M (2012). Fitness costs associated with insecticide resistance. Pest Management Science.

[49] Rabinovich JE (1972). Vital statistics of Triatominae (Hemiptera: Reduviidae) under laboratory conditions. I. Triatoma infestans Klug. J. Med. Entomol..

[50] Khan HA, Shad SA, Akram W (2012). Effect of livestock manures on the fitness of house fly, Musca domestica L. (Diptera: Muscidae). Parasitol. Res..

[51] Khan HAA, Akram W, Arshad M, Hafeez F (2016). Toxicity and resistance of field collected Musca domestica (Diptera: Muscidae) against insect growth regulator insecticides. Parasitol. Res..

[52] Radford P (1967). Growth analysis formulae-their use and abuse. Crop Science.

[53] Yasoob, H., Ali Khan, H. A. & Zhang, Y. Toxicity and Sublethal Effects of Cantharidin on Musca domestica (Diptera: Muscidae). *J*. *Econ*. *Entomol*., tox205 (2017). 10.1093/jee/tox205 29029163

